# Patterns of hepatitis B virus *S* gene escape mutants and reverse transcriptase mutations among genotype D isolates in Jordan

**DOI:** 10.7717/peerj.6583

**Published:** 2019-03-08

**Authors:** Nidaa A. Ababneh, Malik Sallam, Doaa Kaddomi, Abdelrahman M. Attili, Isam Bsisu, Nadia Khamees, Amer Khatib, Azmi Mahafzah

**Affiliations:** 1 Cell Therapy Center (CTC), University of Jordan, Amman, Jordan; 2 Department of Pathology, Microbiology and Forensic Medicine, School of Medicine, University of Jordan, Amman, Jordan; 3 Department of Clinical Laboratories and Forensic Medicine, Jordan University Hospital, Amman, Jordan; 4 Department of Translational Medicine, Faculty of Medicine, Lund University, Malmö, Sweden; 5 Gastroenterology and Liver Division, Department of Internal Medicine, Jordan University Hospital, Amman, Jordan; 6 School of Medicine, University of Jordan, Amman, Jordan

**Keywords:** Hepatitis B, HBV, Mutation, Phylogeny, Epidemiology

## Abstract

**Background:**

Hepatitis B virus (HBV) is an important infectious cause of morbidity and mortality in Jordan. HBV genotype D is the most prevalent in the country. Virus escape mutants in the HBV *S* region is an important public health problem halting preventive efforts. The aim of the current study was to investigate patterns of HBV escape and resistance mutations and to assess domestic transmission of the virus.

**Methods:**

Patients infected with HBV were recruited at Jordan University Hospital (*n* = 56) and were diagnosed during (1984–2012). A total of 37 partial HBV *S* sequences were generated using Sanger’s method. Mutation analysis was done using the HIV grade HBV drug resistance interpretation online tool and Geno2pheno (HBV) online tools. Domestic transmission of HBV was assessed using maximum likelihood phylogenetic inference with similar GenBank sequences.

**Results:**

Genotyping revealed an exclusive presence of sub-genotype D1. Typical HBV escape mutants were identified in seven patients. These mutations included: L109R, Q129R, M133L, S143L and D144E with overall prevalence of 18.9% (95% CI [9.5–34.2]). Reverse transcriptase (RT) sequence analysis showed mutations in three patients with overall prevalence of 8.1% (95% CI [2.8–21.3]). RT mutations included: V173L, S202I, L180M, M204V and T184A. Transmission cluster analysis revealed a relatively high proportion of infections taking place as a result of domestic spread (29.7%).

**Conclusions:**

Based on our findings, RT mutation analysis appears to be of high value before the initiation of therapy in patients with chronic HBV infection in Jordan. Phylogenetic analyses revealed a considerable proportion of local spread in the country which should be considered in the preventive infection control efforts.

## Introduction

The infection by hepatitis B virus (HBV) is considered a major cause of hepatic-related morbidity and mortality globally with an estimated 257 million people living with chronic infection and approximately 887,000 HBV-related deaths by the end of 2015 (World Health Organization, 2017).

The distinguished features of HBV among other human viruses can be summarized as follows: HBV genome is a partially double-stranded circular DNA of extremely small size (about 3,300 bases) with overlapping open reading frames. In addition, HBV replication cycle is peculiar in respect of an intermediate step involving an error-prone reverse transcriptase (RT), and is manifested in a faster evolution compared to other DNA viruses (Fares & Holmes, 2002; Zhou & Holmes, 2007). The high evolutionary rate of HBV have resulted in its diversification in the human population with emergence of several genotypes (A–J), based on inter-genotype sequence variation of more than 8% (Okamoto et al., 1988; Schaefer, 2007; Stuyver et al., 2000; Tatematsu et al., 2009; Yu et al., 2010). The HBV genotypes are further split into subgenotypes designated by numbers based on intra-genotype sequence variation of 4–8% (Norder et al., 2004). Moreover, recombination events have expanded the genetic diversity of HBV with evidence of substantial influence on shaping the evolutionary history of the virus (Simmonds & Midgley, 2005).

The unique features of HBV genome, particularly for the wide spread of overlapping open reading frames, have implications on treatment and prevention of the virus spread (Liang, 2009). This is particularly evident as follows: mutations arising in the RT region as a result of selective pressure by antiviral drugs can end up in the emergence of virus escape mutants in the adjacent *S* region with subsequent lack of response to HBV vaccine (Caligiuri et al., 2016; Croagh, Desmond & Bell, 2015). Among the most commonly described mutations in the RT region are mapped at the codons I169, L180, A181, T184, S202, M204, N236 and M250 (Locarnini & Yuen, 2010). These mutations can arise due to inadequate HBV therapy particularly with drugs that have low genetic barrier for resistance (lamivudine; 3TC) with some mutations having enhanced replication and competence effect on the virus (Bock et al., 2002; Locarnini & Yuen, 2010; Luber, 2005).

It is now confirmed by several reports (Lin et al., 2005; Luongo et al., 2015; Ye, Shang & Li, 2015), that transmission of HBV can occur despite successful vaccination as evidenced by the persistence of hepatitis B surface antibody (anti-HBs) in the patients. In addition, mutant viruses may evade serologic detection by the currently used enzyme immunoassays (Sheldon & Soriano, 2008; Teo & Locarnini, 2010). This can end up in the occurrence of occult hepatitis B infection (Purdy, 2007). On the other hand, a few studies indicated that the public health concerns of escape mutants might not be as previously feared (Leong, Lin & Nguyen, 2016). The prototypic and most stable vaccine-escape mutant is G145R (Hsu et al., 2004; Purdy, 2007). Among other commonly detected vaccine-escape mutants are: P120Q, Q129H, F134Y/L, S143L and D144A/E (Coppola et al., 2015).

In Jordan, the sero-prevalence of chronic HBV was estimated to range between 1.4% and 3.5% indicating a low-intermediate endemicity of the virus (Batayneh & Bdour, 2002; Hamoudi, Ghazzawi & Hamoudi, 2016; Hayajneh, Masaadeh & Hayajneh, 2010). Few studies investigated the risk factors for HBV acquisition in the country and the results pointed to horizontal familial transmission, unhygienic dental care, long-term hemodialysis and living abroad for at least 1 year as the risk factors most frequently associated with the spread of the virus (Al Hijazat & Ajlouni, 2008; Hayajneh, Masaadeh & Hayajneh, 2010). Similar to countries in the Middle East and North Africa (MENA) region, genotype D appeared to be predominating HBV genotypes in Jordan despite the low number of studies investigating this question (Hamoudi, Ghazzawi & Hamoudi, 2016; Lin & Kao, 2015; Masaadeh, Hayajneh & Alqudah, 2008).

Phylogenetic inference can be used to investigate the proportion of domestic spread of viruses and to test hypotheses related to variables that are associated with higher likelihood of transmission (Lin et al., 2005; Pybus & Rambaut, 2009; Sallam et al., 2017).

The aim of the current study was to investigate the patterns of HBV *S* gene escape mutants and the antiviral drug resistance mutations among HBV isolates in Jordan. In addition, we aimed to investigate possible risk factors and proportion of local transmission of the virus in the country.

## Methods

### Study population

A total of 76 individuals with chronic HBV infection that were diagnosed between 1984 and 2012 were included in the study. Of those, 56 were followed-up completely till the study closure and provided serum samples. Diagnosis of chronic infection was defined as the presence of hepatitis B surface antigen (HBsAg) for more than six months. Serum samples were collected between 2010 and 2012. Demographic and clinical data were collected and included information on age, gender, year of diagnosis, possible risk factor(s), antiviral drug treatment status and the viral load. The year of study closure was 2012.

### Ethical permission

The study was approved by the Jordan University Hospital ethical review board (IRB/13/2010) in accordance with the declaration of Helsinki. An informed consent was obtained from all study subjects.

### Viral DNA extraction, amplification and sequencing

HBV DNA was extracted from serum using a QIAamp DNA blood mini kit (QIAGEN, Hilden, Germany). Spin columns were loaded with 200 μL of serum. Following treatment of samples according to the manufacturer’s protocol, HBV DNA was eluted in 40 μL of HyPure water (Thermo Fisher Scientific, Waltham, MA, USA). The *S* region was amplified using primers P1 (nucleotide positions: 1240–1260, 5′-GCGCTGCAGAAGGTTTGTGGCTCCTCTG-3′) and P2 (nucleotide positions: 1928–1948, 5′-GAGTAACTCCACAGTAGCTCC-3′) for the polymerase chain reaction (PCR). The PCR details were as follows: 95 °C for 2 min to activate the enzyme, followed by 35 cycles (denaturation at 95 °C for 30 s, primer annealing at 56 °C for 30 s and elongation 72 °C for 35 s) and finally 10 min at 72 °C. The amplification reaction was performed using a total volume of 25 μL with three μL of DNA template from the extraction in addition to 22 μL of the reaction mix (DNase/RNase free H_2_O 14.4 μL + GoTaq Reaction Buffer 5.0 μL + primer 1 (P1, 100 ng/μL) 1.0 μL + primer 2 (P2, 100 ng/μL) 1.0 μL + 10 μM dNTPs 0.5 μL + GoTaq DNA Polymerase (5U/μL) 0.1 μL using a locally optimized protocol modified from the manufacturer’s instructions) (Promega, Madison, WI, USA). A volume of 1.0 μL of the final product was taken for electrophoresis using 1% agarose gel for evaluation of suitability of DNA for sequencing. The expected amplicon size was 700 bp. Prior to sequencing, all PCR products were purified. To ensure a DNA concentration ideal for sequencing, the purified products were titrated by Nanodrop. Sequencing was performed with primers P1 and P2 using a BigDye Terminator v1.1 Cycle Sequencing Kit (Applied Biosystems, Foster City, CA, USA) according to manufacturer’s instructions through Macrogen commercial sequencing facility (http://foreign.macrogen.co.kr/eng/). Sequence material was assembled using BioEdit software.

### Virus genotyping and mutation analysis

Genotyping was done using HBV database genotyping tool available online (https://hbvdb.ibcp.fr/HBVdb/). Genotype assignments were confirmed using GenBank BLAST tool and the HBVseq tool from the HIV Drug Resistance Database (Shafer, 2006). Escape mutant analysis and drug resistance analysis were conducted using HIV-GRADE HBV drug resistance interpretation online tool and Geno2pheno (HBV) online tool (Neumann-Fraune et al., 2014; Obermeier et al., 2012).

### Phylogenetic analysis of possible transmission links

Phylogenetic inference of possible links among the Jordanian sequences was performed using the maximum likelihood (ML) approach as implemented in PhyML 3.0 (Guindon et al., 2010). A search for similar HBV GenBank sequences was done using the BLAST tool, with retention of the best 10 target sequences. The redundant sequences were removed to exclude potential multiple intra-patient sequences, using the Skipredundant tool from EMBOSS package with 0.98 as the similarity cut-off (Rice, Longden & Bleasby, 2000). The final dataset comprising the Jordanian sequences with similar reference sequences was subjected to five runs of ML analysis using the GTR + G + I nucleotide substitution model with an estimated proportion of invariable sites (*p* = 0.695). Statistical support of the nodes in phylogenetic trees was estimated using the approximate Likelihood Ratio Test Shimodaira-Hasegawa like (aLRT-SH) with 0.85 as the significance level (Anisimova et al., 2011). The ML tree with the highest likelihood was retained for subsequent analysis.

### Statistical analysis

The 95% confidence interval of the prevalence (Wilson score interval, binomial distribution) was calculated using EpiTools epidemiological calculator available online (http://epitools.ausvet.com.au).

### Sequence accession numbers

A total of 37 sequences analyzed in this study were deposited in GenBank. These sequences were assigned with the following accession numbers: MK033359–MK033395.

## Results

### Characteristics of the study population

Out of 76 subjects who were initially enrolled in the study, 56 study subjects who were diagnosed with HBV infection at Jordan University Hospital during 2010–2012, had serum samples and were included for subsequent analysis. The characteristics of the study subjects are illustrated in (Table 1). A total of 75% of the study subjects were males. The median age at the time of diagnosis was 36 years (mean: 39 years, range: 11–77 years). The median time between diagnosis and sampling was 24 months (range: 1–312 months, with three study subjects lacking information).

**Table 1:** Characteristics of the hepatitis B virus (HBV) infected individuals included in the study.

Characteristic	All	
	*n*^1^	%
Total	56	
Sex		
Male	42	75
Female	14	25
Possible risk factor^2^		
Familial	32	57
Blood transfusion	6	11
IDU	3	5
Unknown	15	27
Governorate		
Amman	25	45
Balqa	11	20
Zarqa	9	16
Madaba	3	5
Ma’an	3	5
Karak	3	5
Mafraq	1	2
Unknown	1	2

**Notes:**

1 *n*, number.

2 IDU, injection drug use.

### Determination of HBV genotype

The HBV RT sequences that were utilized in our study were generated using the Sanger population sequencing method. We were able to successfully obtain a total of 37 sequences (66% success rate) with open reading frames each having a final length of 573 bp (RT domain codons: 25–217; HBs antigen (SHB protein) codons: 17–208). All Jordanian HBV sequences were of genotype D using the various genotyping analysis tools. Further analysis based on Geno2pheno (HBV) 2.0 and GenBank blast tools revealed that all Jordanian sequences were of sub-genotype D1. Of the 37 HBV sequences characterized in our study, 10 sequences (27%) were retrieved from individuals who were treatment-naïve, 10 sequences (27%) were retrieved from individuals with chronic HBV infection that underwent treatment.

### RT mutation analysis

Using the Geno2pheno HBV drug resistance tool and HIV grade HBV drug resistance interpretation tool, three patients (8.1% (95% CI [2.8–21.3])), were found to harbor at least a single RT mutation conferring possible or confirmed resistance to RT inhibitors. The details of these mutations are in (Table 2). The first patient was a male with history of treatment with lamivudine who had mutations at established drug resistance positions: L180M, T184A, M204V which confer resistance to lamivudine, entecavir and telbivudine. The second patient was a female with unknown history of treatment who had mutations at established drug resistance positions: L180M, M204V which confer resistance to lamivudine, telbivudine and partial resistance to entecavir. The third patient was a male with history of treatment with lamivudine who had mutations at established drug resistance positions: V173L, L180M, M204V which confer resistance to lamivudine, telbivudine and partial resistance to entecavir.

**Table 2:** The distribution of reverse transcriptase (RT) mutations and HBsAg vaccine escape mutations among the study population.

Sample ID	Age	Gender	Year of Dx^1^	Sub-genotype	Rx status^2^	Mutations RT^3^	Escape mutations
22	38	Male	2009	D1	Naïve	N53S, F122I, H124D, Y135S, H216L, L217F	–
39	35	Male	2009	D1	Unknown	Y135S, H216L, L217F	–
84	35	Male	Unknown	D1	Unknown	F122I, H124Y, Y135S, H216L, L217F	–
97	36	Male	1995	D1	Naïve	R110G, Y135S, H216L	–
23	68	Male	2009	D1	Unknown	F122I, H124Y, Y135S, H216L	–
66	38	Male	2004	D1	Unknown	H124Y, Y135S, I163V, H216I, L217F	–
85	33	Female	2004	D1	Naïve	S119P, H124Y, Y135S, H216L	–
99	33	Male	2007	D1	Experienced (Lamivudine)	F122I, H124Y, Y135S, L217F	–
4	30	Male	2006	D1	Naïve	L91I, H124Y, N131D, Y135S, H216L, L217F	–
24	22	Female	2009	D1	Experienced (Lamivudine)	Y135S, H216I, L217F	–
45	38	Female	1998	D1	Experienced (Lamivudine)	H124Y, Y135S, Q215H, H216L	–
101	30	Male	2010	D1	Unknown	F122I, H124Y, Y135S, H216L, L217F	–
2	60	Male	2009	D1	Experienced (Lamivudine)	Y135S, H216L, L217F	R122K
46	35	Female	2000	D1	Experienced (Lamivudine)	H124Y, Y135S, Q215H, H216F, L217W	–
57	39	Female	2010	D1	Unknown	A38E, Y54H, M129L, Y135S, V173L, H216L	–
87	30	Male	2011	D1	Unknown	K32M, Y135S, S213T, H216I	–
106	47	Male	2011	D1	Unknown	F122I, H124Y, Y135S, H216L	–
6	13	Male	2009	D1	Experienced (Lamivudine)	H124Y, Y135S, Q149K, H216L	–
49	32	Female	2008	D1	Unknown	S85A, Y135S, N139D, L199V, H216I, L217W	L109R
76	51	Female	2009	D1	Naïve	L91I, H124D, Y135S, K212N, H216P	S143L
81	38	Male	1998	D1	Experienced (Lamivudine)	S78T, F122I, H124Y, Y135S, H216I	–
83	51	Male	2000	D1	Unknown	A38E, R110G, F122I, H124Y, Y135S, Q149K, S176T, S185I, H197P, C198R, S202I, V208I, A211G, K212G, S213Q, V214Y, Q215L, H216L, L217F	–
5	11	Male	2009	D1	Experienced (Lamivudine)	Y135S, H216L, L217F	–
34	26	Male	2008	D1	Unknown	F122I, H124Y, Y135S, H216L	–
52	25	Male	2009	D1	Naïve	H124Y, Y135S, H216P	–
70	44	Male	2011	D1	Naïve	Y54G, N76D, Y135S, Y141F, H216I, L217F	M133L
89	24	Male	2009	D1	Unknown	Y54H, Y135S, H216L	D144E
105	48	Female	2011	D1	Naïve	H124Y, T128P, Y135S, L145M, H216P	–
8	60	Male	1984	D1	Experienced (Lamivudine)	A38E, Y54H, H124Y, M129L, Y135S, **V173L**, **L180M**, **M204V**, H216L	–
35	29	Male	2009	D1	Unknown	S109P, Y135S, H216L, L217F	–
56	36	Male	2010	D1	Unknown	T37A, F122L, N123D, Y135S, H216L	–
77	65	Male	2011	D1	Naïve	H124Y, Y135S, G152K, K154N, I187L, H216P, L217F	–
103	23	Female	2011	D1	Naïve	N53K, F122I, H124Y, Y135S, H216L	–
63	77	Male	2004	D1	Unknown	H124Y, Y135S, Q215H, H216T, L217F	R122K
38	34	Female	2007	D1	Unknown	F122I, H124Y, Y135S, H216I, L217W	–
58	65	Female	2006	D1	Unknown	V27A, A38E, S117N, N118D, H124D, Y135S, S137T, **L180M**, **M204V**, H216L	R122K, Q129R
104	37	Male	2007	D1	Experienced (Lamivudine)	A38E, H124Y, Y135S, **L180M**, **T184A**, **M204V**, H216I	–

**Notes:**

RT mutation conferring possible or confirmed resistance to RT inhibitors are highlighted in bold.

1 Dx, diagnosis.

2 Rx, treatment.

3 RT, reverse transcriptase.

### HBsAg escape mutations

The overall prevalence of typical HBsAg escape mutants was 18.9% (95% CI [9.5–34.2]). The R122K mutation which affects HBV detection was found in three patients. The L109R mutation that is a vaccine-escape mutant was found in a single patient. The S143L mutation which is both a vaccine-escape mutant and affects HBV detection was found in a single patient. The M133L and D144E mutations that are vaccine-escape mutants, affecting HBV detection and immunoglobulin therapy were found each in a single patient. Finally, a single patient had Q129R mutation that is a vaccine-escape mutant and affects HBV detection besides the R122K mutation.

### Possible domestic transmission of HBV in Jordan

**Figure 1:**
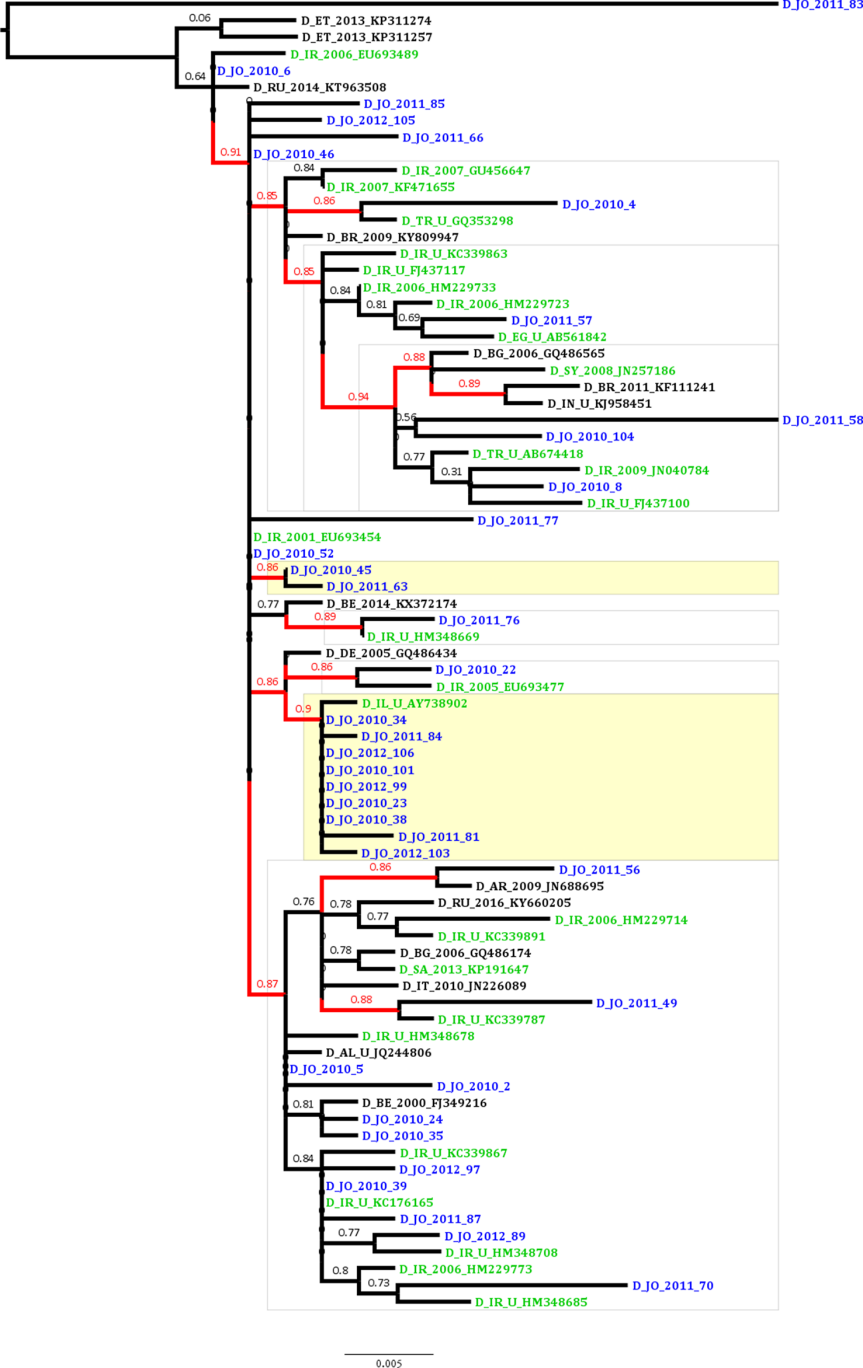
Maximum likelihood tree of the Jordanian HBV sequences with similar GenBank sequences. The Jordanian sequence names are highlighted in blue color. The statistically supported branches (with approximate Likelihood Ratio Test Shimodaira-Hasegawa like values ≥ 0.85) are highlighted in red color. The GenBank sequences collected in the Middle East and North Africa (MENA) region are highlighted in green color.

To estimate the proportion of HBV sequences that are possibly linked in transmission clusters indicating domestic transmission, ML analysis was conducted which revealed that the proportion of phylogenetic clustering was 29.7% (11 of the 37 Jordanian sequences; Fig. 1). The clustering sequences were distributed among a dyad (two sequences) and a network of 10 sequences (with nine Jordanian sequences and a single Israeli sequence). The non-clustering Jordanian sequences were present in supported monophyletic clades together with sequences that were collected in Iran, Turkey, Syria and Egypt (Fig. 1).

## Discussion

**Figure 2:**
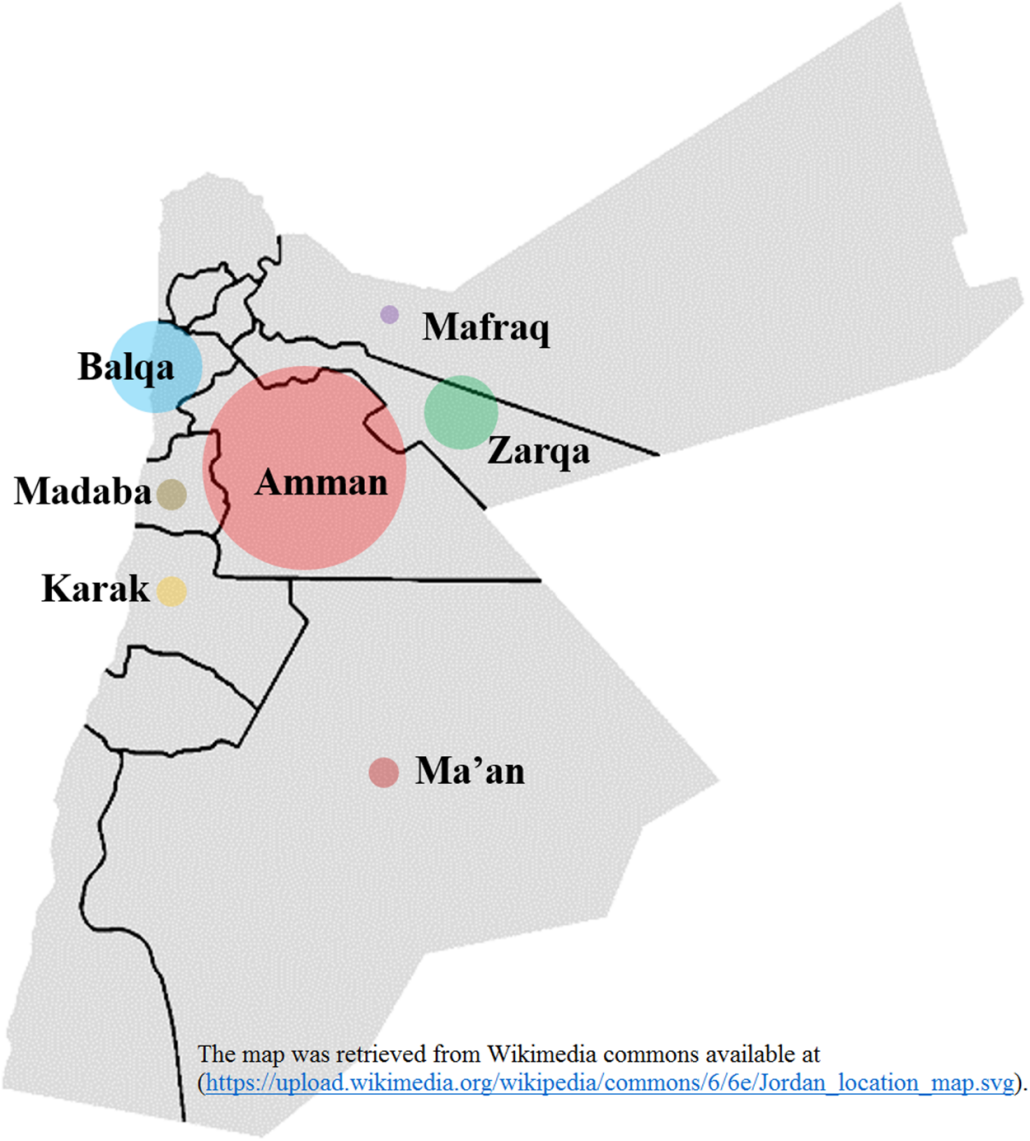
Hepatitis B virus sample distribution from different Jordanian governorates. The diameter of each circle is proportional to the number of samples; Amman: 25, Balqa: 11, Zarqa: 9, Madaba: 3, Ma’an: 3, Karak: 3, Mafraq: 1. The map was retrieved from Wikimedia commons available at: (https://upload.wikimedia.org/wikipedia/commons/6/6e/Jordan_location_map.svg).

The epidemiological and antiviral drug resistance data of HBV in Jordan are limited. The results of our study are strengthened by the good coverage of sampling from different regions in the country (Fig. 2). Genotyping results showed that subgenotype D1 was found exclusively, which is consistent with previous studies from Jordan and the MENA region where this sub-genotype predominates (Abdelnabi et al., 2014; Al Baqlani et al., 2014; Asaad et al., 2015; Ciccozzi et al., 2014; El-Mowafy et al., 2017; Hamoudi, Ghazzawi & Hamoudi, 2016; Ziaee et al., 2016). The finding that all HBV isolates in our study were exclusively of genotype D is possibly related to origins of infections in the MENA in which genotype D predominates. This is supported by the finding of intermingling of sequences from Iran, Turkey, Syria and Saudi Arabia (Fig. 1).

In the current study, we investigated the patterns of HBsAg escape mutants and HBV RT mutations for the first time in Jordan to the best of our knowledge. The prevalence of HBsAg escape mutants in Jordan was 18.9%, with the R122K mutation which affects HBV detection being the most prevalent type. This is comparable to a study done in Iran, which showed that the frequency of 14% major hydrophilic region mutations (MHR), with the most frequent ones of P120T/S and R122K/T (Moradi et al., 2012). In Turkey, the most frequent mutations observed were T143M and K122R, whereas in Egypt 14.8% presented with mutations in the MHR, and eight different mutations were discovered: R122K, S143L, L109P, S114P, S117N, P127S, P127T and Y134F (Zeid, Ramadan & Shemis, 2016). Previous studies have shown the significance of R122 as a serologic determinant for HBV subtype, with subsequent effect on the antigenicity and immunogenicity of the virus (Ashton-Rickardt & Murray, 1989; Hou et al., 2001; Wu et al., 2012).

The mutations in the HBV RT have been shown to affect the management of HBV infection (Caligiuri et al., 2016; Terrault et al., 2016). For instance, the patients in our study sample who developed HBV RT mutations showed complete or partial resistance to treatment by lamivudine, telbivudine and entecavir. In the three patients with lamivudine resistance mutations, partial or complete resistance to entecavir was detected which is likely related to selection of HBV entecavir mutants in those patients. This finding is in line with recent findings by Geipel et al. (2015).

The most common resistance mutations in Jordan were 180M and 204V, which were always present as co-mutations. Other identified mutations were 173L and 184A mutations, and they were co-mutated with 180M, 204V. M204I/V mutations are frequently accompanied by compensatory mutations in other domains such as 59 rtV173L, rtL180M, rtT184S/G, 58 rtI169T, rtS202I, rtL80V/I and rtQ215S which enhanced the replication efficiency of rt204I/V mutants without significantly affecting lamivudine resistance, by compensating the decrease in efficiency due to resistance-associated changes (Bartholomeusz & Locarnini, 2006a, 2006b; Caligiuri et al., 2016; Zoulim & Locarnini, 2009). For example, dual rtL180M and M204V/I mutants were frequently found in patients in Italy, with three patients having triple mutation of rtV173L, rtL180M and M204V (Quiros-Roldan et al., 2008). Upon comparing the mutant strains with wild HBV strains, patients with rtM204V/I more frequently presented with severe acute hepatitis B and lower serum HBV DNA values (Coppola et al., 2013).

A recent meta-analysis has shown that the incidence of spontaneous primary and secondary mutations among untreated chronic hepatitis B patients was 4.9% for primary mutations of rtM204V/I, while the natural incidence of secondary rtL180M mutations was 2.7% (Zhang et al., 2015). Previous literature suggests that lamivudine is the main cause of YMDD (tyrosine-methionine-aspartateaspartate) mutations (M204I/V) within the catalytic sites (C domain) in HBV P-ORF (Caligiuri et al., 2016; Ji et al., 2011). In Turkey, Japan and China, the a high rates of spontaneous YMDD mutation may increase the unresponsiveness to lamivudine, leading to prolonged therapy time and extra cost (Zhang et al., 2015), for which we need to perform further studies to investigate the probability of development of antiviral resistant mutants worldwide, and to study the effects of the infection with mutant viruses on the clinical course of the disease.

Upon conducting ML analysis to investigate the possibility of domestic transmission of HBV in Jordan, the proportion of phylogenetic clustering was 29.7%. Horizontal transmission is the main mode of transmission of HBV in Jordan (André, 2000). Clustering of chronic HBV infections within the family is common in areas of where HBV is endemic, and horizontal transmission during early childhood is a major route of HBV transmission (Dumpis et al., 2001; Lin et al., 2005; Zampino et al., 2002). The high proportion of clustering among the study subjects highlights the need for intervention measures by public health control strategies to stop the chains of forward transmission of the virus.

Several caveats of our study should be addressed including the low sampling coverage and the short length of RT sequences that were used to conduct the transmission cluster analysis. The study was closed in 2012, which highlights the need for a follow up study to assess the recent trends of genotype and risk factors for HBV infection in the country. Another caveat of our study is the lack of clinical and serological data from the study subjects.

## Conclusions

To conclude, we investigated the genotype distribution, risk factors, RT and HBsAg vaccine escape mutations and the transmission of HBV in Jordan over a large area of the country. Based on the findings of our study, we recommend RT mutation analysis before the initiation of therapy in patients with chronic HBV infection in Jordan. Lamivudine monotherapy is strongly discouraged due to high risk of resistance development. In addition, the relatively high proportion of phylogenetic clustering highlights the need for public health control measures to prevent forward transmission of the virus in the country and regionally.

## Supplemental Information

10.7717/peerj.6583/supp-1 Supplemental Information 1 Nucleotide HBV sequences that were generated for the study.

## Additional Information and Declarations

### Data Availability

The following information was supplied regarding data availability: A complete list of the nucleotide sequences that were generated for the study are available in Supplemental File 1.

## References

[ref-1] Abdelnabi Z, Saleh N, Baraghithi S, Glebe D, Azzeh M (2014). Subgenotypes and mutations in the S and polymerase genes of hepatitis B virus carriers in the West Bank, Palestine. PLOS ONE.

[ref-2] Al Baqlani SA, Sy BT, Ratsch BA, Al Naamani K, Al Awaidy S, Busaidy SA, Pauli G, Bock CT (2014). Molecular epidemiology and genotyping of hepatitis B virus of HBsAg-positive patients in Oman. PLOS ONE.

[ref-3] Al Hijazat M, Ajlouni YM (2008). Hepatitis B infection among patients receiving chronic hemodialysis at the Royal Medical Services in Jordan. Saudi Journal of Kidney Diseases and Transplantation.

[ref-4] André F (2000). Hepatitis B epidemiology in Asia, the middle East and Africa. Vaccine.

[ref-5] Anisimova M, Gil M, Dufayard JF, Dessimoz C, Gascuel O (2011). Survey of branch support methods demonstrates accuracy, power, and robustness of fast likelihood-based approximation schemes. Systematic Biology.

[ref-6] Asaad AM, Al-Ayed MS, Aleraky M, Qureshi MA (2015). Hepatitis B virus genotyping in chronic hepatitis B patients in southwestern Saudi Arabia. Brazilian Journal of Infectious Diseases.

[ref-7] Ashton-Rickardt PG, Murray K (1989). Mutations that change the immunological subtype of hepatitis B virus surface antigen and distinguish between antigenic and immunogenic determination. Journal of Medical Virology.

[ref-8] Bartholomeusz A, Locarnini S (2006a). Hepatitis B virus mutations associated with antiviral therapy. Journal of Medical Virology.

[ref-9] Bartholomeusz A, Locarnini SA (2006b). Antiviral drug resistance: clinical consequences and molecular aspects. Semin Liver Dis.

[ref-10] Batayneh N, Bdour S (2002). Risk of perinatal transmission of hepatitis B virus in Jordan. Infectious Diseases in Obstetrics and Gynecology.

[ref-11] Bock CT, Tillmann HL, Torresi J, Klempnauer J, Locarnini S, Manns MP, Trautwein C (2002). Selection of hepatitis B virus polymerase mutants with enhanced replication by lamivudine treatment after liver transplantation. Gastroenterology.

[ref-12] Caligiuri P, Cerruti R, Icardi G, Bruzzone B (2016). Overview of hepatitis B virus mutations and their implications in the management of infection. World Journal of Gastroenterology.

[ref-13] Ciccozzi M, Ciccaglione AR, Lo Presti A, Equestre M, Cella E, Ebranati E, Gabanelli E, Villano U, Bruni R, Yalcinkaya T, Tanzi E, Zehender G (2014). Evolutionary dynamics of HBV-D1 genotype epidemic in Turkey. Journal of Medical Virology.

[ref-14] Coppola N, Onorato L, Minichini C, Di Caprio G, Starace M, Sagnelli C, Sagnelli E (2015). Clinical significance of hepatitis B surface antigen mutants. World Journal of Hepatology.

[ref-15] Coppola N, Tonziello G, Colombatto P, Pisaturo M, Messina V, Moriconi F, Alessio L, Sagnelli C, Cavallone D, Brunetto M (2013). Lamivudine-resistant HBV strain rtM204V/I in acute hepatitis B. Journal of Infection.

[ref-16] Croagh CM, Desmond PV, Bell SJ (2015). Genotypes and viral variants in chronic hepatitis B: a review of epidemiology and clinical relevance. World Journal of Hepatology.

[ref-17] Dumpis U, Holmes EC, Mendy M, Hill A, Thursz M, Hall A, Whittle H, Karayiannis P (2001). Transmission of hepatitis B virus infection in Gambian families revealed by phylogenetic analysis. Journal of Hepatology.

[ref-18] El-Mowafy M, Elgaml A, El-Mesery M, Elegezy M (2017). Molecular analysis of Hepatitis B virus sub-genotypes and incidence of preS1/preS2 region mutations in HBV-infected Egyptian patients from Mansoura. Journal of Medical Virology.

[ref-19] Fares MA, Holmes EC (2002). A revised evolutionary history of hepatitis B virus (HBV). Journal of Molecular Evolution.

[ref-20] Geipel A, Seiz PL, Niekamp H, Neumann-Fraune M, Zhang K, Kaiser R, Protzer U, Gerlich WH, Glebe D, Consortium H (2015). Entecavir allows an unexpectedly high residual replication of HBV mutants resistant to lamivudine. Antiviral Therapy.

[ref-22] Guindon S, Dufayard JF, Lefort V, Anisimova M, Hordijk W, Gascuel O (2010). New algorithms and methods to estimate maximum-likelihood phylogenies: assessing the performance of PhyML 3.0. Systematic Biology.

[ref-23] Hamoudi W, Ghazzawi I, Hamoudi MMY (2016). Hepatitis B genotypic and serologic characteristics in Jordan. Journal of the Royal Medical Services.

[ref-24] Hayajneh WA, Masaadeh HA, Hayajneh YA (2010). A case-control study of risk factors for hepatitis B virus infection in North Jordan. Journal of Medical Virology.

[ref-25] Hou J, Wang Z, Cheng J, Lin Y, Lau GK, Sun J, Zhou F, Waters J, Karayiannis P, Luo K (2001). Prevalence of naturally occurring surface gene variants of hepatitis B virus in nonimmunized surface antigen–negative Chinese carriers. Hepatology.

[ref-26] Hsu HY, Chang MH, Ni YH, Chen HL (2004). Survey of hepatitis B surface variant infection in children 15 years after a nationwide vaccination programme in Taiwan. Gut.

[ref-27] Ji D, Liu Y, Si L-L, Li L, Chen G-F, Xin S-J, Zhao J-M, Xu D (2011). Variable influence of mutational patterns in reverse-transcriptase domain on replication capacity of hepatitis B virus isolates from antiviral-experienced patients. Clinica Chimica Acta.

[ref-28] Leong J, Lin D, Nguyen MH (2016). Hepatitis B surface antigen escape mutations: indications for initiation of antiviral therapy revisited. World Journal of Clinical Cases.

[ref-29] Liang TJ (2009). Hepatitis B: the virus and disease. Hepatology.

[ref-31] Lin CL, Kao JH (2015). Hepatitis B virus genotypes and variants. Cold Spring Harbor Perspectives in Medicine.

[ref-32] Lin C-L, Kao J-H, Chen B-F, Chen P-J, Lai M-Y, Chen D-S (2005). Application of hepatitis B virus genotyping and phylogenetic analysis in intrafamilial transmission of hepatitis B virus. Clinical Infectious Diseases.

[ref-33] Locarnini SA, Yuen L (2010). Molecular genesis of drug-resistant and vaccine-escape HBV mutants. Antiviral Therapy.

[ref-34] Luber AD (2005). Genetic barriers to resistance and impact on clinical response. Journal of the International AIDS Society.

[ref-35] Luongo M, Critelli R, Grottola A, Gitto S, Bernabucci V, Bevini M, Vecchi C, Montagnani G, Villa E (2015). Acute hepatitis B caused by a vaccine-escape HBV strain in vaccinated subject: sequence analysis and therapeutic strategy. Journal of Clinical Virology.

[ref-36] Masaadeh HA, Hayajneh WA, Alqudah EA (2008). Hepatitis B virus genotypes and lamivudine resistance mutations in Jordan. World Journal of Gastroenterology.

[ref-37] Moradi A, Zhand S, Ghaemi A, Javid N, Tabarraei A (2012). Mutations in the S gene region of hepatitis B virus genotype D in Golestan Province-Iran. Virus Genes.

[ref-38] Neumann-Fraune M, Beggel B, Kaiser R, Obermeier M (2014). Hepatitis B virus drug resistance tools: one sequence, two predictions. Intervirology.

[ref-39] Norder H, Courouce AM, Coursaget P, Echevarria JM, Lee SD, Mushahwar IK, Robertson BH, Locarnini S, Magnius LO (2004). Genetic diversity of hepatitis B virus strains derived worldwide: genotypes, subgenotypes, and HBsAg subtypes. Intervirology.

[ref-40] Obermeier M, Pironti A, Berg T, Braun P, Daumer M, Eberle J, Ehret R, Kaiser R, Kleinkauf N, Korn K, Kucherer C, Muller H, Noah C, Sturmer M, Thielen A, Wolf E, Walter H (2012). HIV-GRADE: a publicly available, rules-based drug resistance interpretation algorithm integrating bioinformatic knowledge. Intervirology.

[ref-41] Okamoto H, Tsuda F, Sakugawa H, Sastrosoewignjo RI, Imai M, Miyakawa Y, Mayumi M (1988). Typing hepatitis B virus by homology in nucleotide sequence: comparison of surface antigen subtypes. Journal of General Virology.

[ref-42] Purdy MA (2007). Hepatitis B virus S gene escape mutants. Asian Journal of Transfusion Science.

[ref-43] Pybus OG, Rambaut A (2009). Evolutionary analysis of the dynamics of viral infectious disease. Nature Reviews Genetics.

[ref-44] Quiros-Roldan E, Calabresi A, Lapadula G, Tirelli V, Costarelli S, Cologni G, Zaltron S, Puoti M, Carosi G, Torti C (2008). Evidence of long-term suppression of hepatitis B virus DNA by tenofovir as rescue treatment in patients coinfected by HIV. Antiviral Therapy.

[ref-45] Rice P, Longden I, Bleasby A (2000). EMBOSS: the European molecular biology open software suite. Trends in Genetics.

[ref-46] Sallam M, Sahin GO, Ingman M, Widell A, Esbjornsson J, Medstrand P (2017). Genetic characterization of human immunodeficiency virus type 1 transmission in the Middle East and North Africa. Heliyon.

[ref-47] Schaefer S (2007). Hepatitis B virus taxonomy and hepatitis B virus genotypes. World Journal of Gastroenterology.

[ref-48] Shafer RW (2006). Rationale and uses of a public HIV drug-resistance database. Journal of Infectious Diseases.

[ref-49] Sheldon J, Soriano V (2008). Hepatitis B virus escape mutants induced by antiviral therapy. Journal of Antimicrobial Chemotherapy.

[ref-50] Simmonds P, Midgley S (2005). Recombination in the genesis and evolution of hepatitis B virus genotypes. Journal of Virology.

[ref-51] Stuyver L, De Gendt S, Van Geyt C, Zoulim F, Fried M, Schinazi RF, Rossau R (2000). A new genotype of hepatitis B virus: complete genome and phylogenetic relatedness. Journal of General Virology.

[ref-52] Tatematsu K, Tanaka Y, Kurbanov F, Sugauchi F, Mano S, Maeshiro T, Nakayoshi T, Wakuta M, Miyakawa Y, Mizokami M (2009). A genetic variant of hepatitis B virus divergent from known human and ape genotypes isolated from a Japanese patient and provisionally assigned to new genotype J. Journal of Virology.

[ref-53] Teo CG, Locarnini SA (2010). Potential threat of drug-resistant and vaccine-escape HBV mutants to public health. Antiviral Therapy.

[ref-54] Terrault NA, Bzowej NH, Chang KM, Hwang JP, Jonas MM, Murad MH (2016). AASLD guidelines for treatment of chronic hepatitis B. Hepatology.

[ref-21] World Health Organization (2017). Global Hepatitis Report.

[ref-55] Wu C, Deng W, Deng L, Cao L, Qin B, Li S, Wang Y, Pei R, Yang D, Lu M, Chen X (2012). Amino acid substitutions at positions 122 and 145 of hepatitis B virus surface antigen (HBsAg) determine the antigenicity and immunogenicity of HBsAg and influence in vivo HBsAg clearance. Journal of Virology.

[ref-56] Ye Q, Shang SQ, Li W (2015). A new vaccine escape mutant of hepatitis B virus causes occult infection. Human Vaccines & Immunotherapeutics.

[ref-57] Yu H, Yuan Q, Ge SX, Wang HY, Zhang YL, Chen QR, Zhang J, Chen PJ, Xia NS (2010). Molecular and phylogenetic analyses suggest an additional hepatitis B virus genotype “I”. PLOS ONE.

[ref-58] Zampino R, Lobello S, Chiaramonte M, Venturi-Pasini C, Dumpis U, Thursz M, Karayiannis P (2002). Intra-familial transmission of hepatitis B virus in Italy: phylogenetic sequence analysis and amino-acid variation of the core gene. Journal of Hepatology.

[ref-59] Zeid WMA, Ramadan DI, Shemis MA (2016). Prevalence of mutations within major hydrophilic region of hepatitis B virus and their correlation with genotypes among chronically infected patients in Egypt. Arab Journal of Gastroenterology.

[ref-60] Zhang Q, Liao Y, Cai B, Li Y, Li L, Zhang J, An Y, Wang L (2015). Incidence of natural resistance mutations in naïve chronic hepatitis B patients: a systematic review and meta-analysis. Journal of Gastroenterology and Hepatology.

[ref-61] Zhou Y, Holmes EC (2007). Bayesian estimates of the evolutionary rate and age of hepatitis B virus. Journal of Molecular Evolution.

[ref-62] Ziaee M, Javanmard D, Sharifzadeh G, Hasan Namaei M, Azarkar G (2016). Genotyping and mutation pattern in the overlapping MHR region of HBV isolates in Southern Khorasan, Eastern Iran. Hepatitis Monthly.

[ref-63] Zoulim F, Locarnini S (2009). Hepatitis B virus resistance to nucleos (t) ide analogues. Gastroenterology.

